# Widespread Encephalitis Following Acute Sinusitis in a Pediatric Patient

**DOI:** 10.1590/0037-8682-0024-2025

**Published:** 2025-06-02

**Authors:** Elif Gozgec, Muhammed Mukremin Bugdaci

**Affiliations:** 1 Ataturk University School of Medicine, Department of Radiology, Erzurum, Turkey.

**FIGURE 1:**
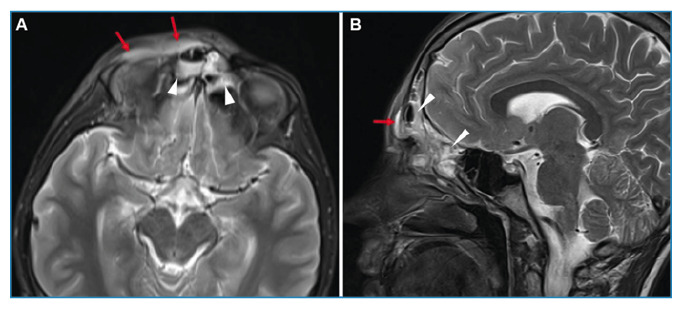
Axial **(A)** and sagittal **(B)** section T2-weighted brain MRI show diffuse intensity increases (arrowheads) of sinusitis in the ethmoid cells and frontal sinus. Additionally, greater intensity and thickness (red arrows) are observed in the right periorbital area.

**FIGURE 2:**
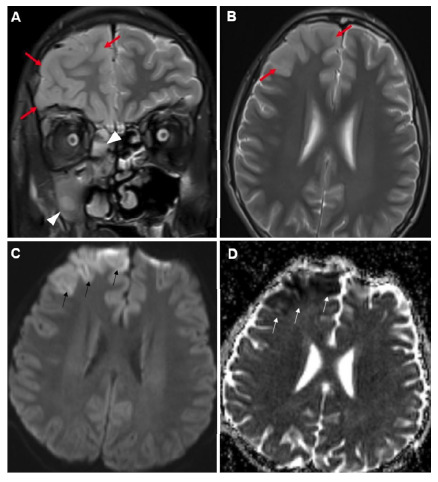
Coronal **(A)** and axial **(B)** section T2-weighted brain MRI scans show greater intensity in the right maxillary sinus and ethmoid cells consistent with sinusitis (arrowheads) and diffuse thickness and greater intensity in the gray matter in the right frontal lobe (red arrows). Axial diffusion-weighted images show greater intensity in the B-1000 series **(C)** and hypointense appearance (arrows) in apparent diffusion coefficient mapping **(D)** in the frontal lobe.

A 16-year-old boy was admitted to our clinic with a complain of headache, fever, rhinorrhea, and erythema of the right eye for 3 days with no known history of disease or surgery. Physical examination revealed tonsillar hyperemia and tenderness in the right frontal region. Hyperemia and ptosis were observed in the right eye. Neurological examinations revealed no abnormalities. The white cell count was 8.7×10^3^/µL; C-reactive protein concentration was 68.6 mg/dL; and sedimentation rate was 64 mm/h. Magnetic resonance imaging (MRI) showed pansinusitis and inflammation signs in the right periorbital region (Figure 1). T2-weighted images revealed greater thickness and intensity in the grey matter of the right frontal lobe of the brain. These areas exhibited marked restrictions on diffusion-weighted images and minimal contrast enhancement (Figure 2). Intravenous ceftriaxone, metronidazole, and vancomycin treatment was initiated under the diagnosis of encephalitis. Controlled MRI performed on the 14th day of treatment revealed significant regression of encephalitis.

Encephalitis, a rare intracranial complication of sinusitis, is associated with high risks of mortality and morbidity^1^. Complications are more common in the frontal region due to the high frequency of frontal sinusitis in adolescents, high blood flow in the frontal sinuses, and the high density of diploic veins in this region^2^. Diagnosis can be difficult owing to the potential for symptoms similar to sinusitis. Radiological imaging is crucial for diagnosis and monitoring.
